# Symptomatic Acute Hepatitis C Infection Following a Single Episode of Unprotected Sexual Intercourse

**DOI:** 10.1155/2016/8639098

**Published:** 2016-11-10

**Authors:** Benjamin Butler, Bhaskar Narayan, Jonathan Potts, Julian Emmanuel

**Affiliations:** Department of Acute Medicine, Royal London Hospital, Barts Health NHS Trust, Whitechapel Road, London E1 1BB, UK

## Abstract

A previously healthy 23-year-old MSM presented with jaundice, systemic upset, and rash 2 months after a single episode of unprotected sexual intercourse. Liver biochemistry was grossly deranged, with markedly elevated transaminases and hyperbilirubinaemia. Serology was positive for genotype 1a hepatitis C virus (HCV) and in the absence of other causes, acute HCV infection was suspected. He was subsequently successfully treated with pegylated interferon and ribavirin for 24 weeks and made a full clinical and biochemical recovery.

## 1. Introduction

Hepatitis C virus (HCV) remains one of the leading causes of chronic liver disease, affecting an estimated 180 million people worldwide and causing 350,000 deaths annually [1]. The acute infection is often clinically mild or asymptomatic and consequently the majority of cases are diagnosed in chronic carriers, who have been infected at some uncertain time in the past. Fifteen percent of individuals experience a viral prodrome and symptomatic hepatitis, including jaundice, dark urine, anorexia, and abdominal pain. Viral clearance occurs in 10–50% of cases and those with symptomatic acute infection are more likely to spontaneously eradicate the virus [2]. Confident diagnosis of acute HCV infection requires evidence of seroconversion. Alternatively, acute HCV hepatitis may be suspected in the setting of a clinical and biochemical picture of acute hepatitis (jaundice with transaminases over tenfold the upper limit of normal) in the context of a recent source of transmission and where other causes of hepatitis are excluded [3].

It is rare for HCV to be acquired through heterosexual intercourse with a regular partner and there is only a slight increase in risk with multiple heterosexual partners [4]. Sexual transmission of hepatitis C in men who have sex with men (MSM) is well recognised, but the literature mainly describes this in HIV-positive men, particularly those who engage in high-risk sexual practices [4, 5]. Routine sexual health screening for blood-borne viruses in this group currently excludes hepatitis C. Screening is considered only in high-risk individuals, specified as those who engage in traumatic sexual behaviour, those who engage in intravenous drug use (IVDU), those who engage in “chem sex,” and those known to be HIV positive or with lymphogranuloma venereum; unprotected sexual intercourse alone is not considered high-risk [6]. Here we present a case to support routine screening for hepatitis C infection in all MSM.

## 2. Case

A 23-year-old Caucasian homosexual man presented to the emergency department with a 9-day history of painless jaundice following two weeks of constitutional upset, associated with an intermittent rash over the abdomen. He reported a single episode of unprotected anoreceptive intercourse two months previously but denied IVDU or foreign travel. His only past medical history was of mild asthma and he denied any recent history of prescribed, over-the-counter, or herbal medications. His nonresolving symptoms prompted a General Practitioner referral for urgent medical review. He had been seen in a genitourinary medicine clinic one month prior to presentation and tested negative for syphilis, gonorrhoea, chlamydia, and HIV. He was an occasional alcohol drinker. Family history was unremarkable.

On examination, the patient was icteric but free from hepatic encephalopathy. He was mildly tachycardic but normotensive and apyrexial. A rash with 1 cm nonblanching macular lesions was evident on the abdomen. His abdomen was soft and nontender, with no guarding, rigidity, detectable organomegaly, or stigmata of chronic liver disease. Other physical examination was unremarkable.

## 3. Investigations

Laboratory investigations showed marked hyperbilirubinaemia with a brisk transaminitis (Table 1) but no coagulopathy.

Viral serology (Table 2) was positive for genotype 1a hepatitis C infection with a viral load of 5.8 log_10_ IU/mL. Screening tests for other infectious, autoimmune, or inherited causes of liver disease were negative. Abdominal ultrasonography showed no biliary duct dilatation or cholelithiasis and patent hepatic and portal vasculature.

## 4. Treatment

Acute hepatitis C was suspected on clinical grounds and the patient was referred to the hepatology service. The acute hepatitis resolved over the following four months (Table 1) but viraemia persisted. Sixteen weeks after diagnosis antiviral treatment was commenced with a 24-week course of pegylated interferon-*α* and ribavirin.

## 5. Outcome

The patient tolerated treatment well and became HCV RNA negative from treatment week four. He ultimately achieved a sustained virological response (SVR), defined by undetectable HCV RNA 24 weeks after completion of antiviral treatment.

## 6. Discussion

Our case is noteworthy for a number of reasons; it is uncommon for HCV to cause a clinical acute hepatitis with transaminases nearly 40 times the upper limit of normal and severe hyperbilirubinaemia. Furthermore, the patient did not have any clear risk factors for blood-borne transmission and although he had a history of recent anoreceptive sexual intercourse with a male partner, he was HIV-negative and did not engage in any behaviours considered to be particularly “high-risk” for HCV transmission. It is of interest that our patient did not have a hepatitis C test as part of his initial genitourinary medicine assessment and this was not available for retrospective testing.

The latest guidelines from the British Association of Sexual Health and HIV (BASHH) state, “*Currently there is no evidence that HIV-negative MSM without other risk factors should be routinely screened”* [6]. The omission of a hepatitis C test in this patient was therefore entirely in keeping with these guidelines. A recent Spanish study suggested that there may be significant HCV sexual transmission rates in HIV-negative MSM in the presence of other risk factors, such as recreational drug use [7]. Indeed, a study in Manchester, United Kingdom, suggested that it is difficult to reliably risk-assess MSM and that perhaps this group should be routinely tested for HCV [8]. Of note are patients receiving preexposure prophylaxis (PrEP) for the prevention of HIV-1 acquisition; increased incidence of HCV transmission has been reported in this group but ongoing testing is not currently recommended [9].

Spontaneous viral clearance occurs in 30–50% of those with symptomatic acute HCV infection, normally within 3 months of the onset of symptoms [10]. For those with persistent viraemia beyond 12 weeks, antiviral treatment for acute HCV should be considered to prevent progression to chronic infection. SVR rates using conventional therapies in acute HCV are superior to those in chronic HCV infection and there are additional public health benefits in reducing the number of potentially infectious individuals among high-risk groups. Until recently interferon-based regimens have been used, with pegylated interferon-*α* monotherapy for 24 weeks leading to SVR in >90% of cases [3]. The addition of ribavirin to pegylated interferon-*α* may further improve SVR rates, particularly in the treatment of HCV genotype 1a [11, 12] and in patients with other predictors of treatment failure, such as slow viral response or HIV coinfection [3]. Our patient received pegylated interferon and ribavirin combination therapy due to the presence of genotype 1a and suspected rather than proven acute HCV infection. Over recent years numerous highly effective oral direct-acting antiviral agents have emerged, which offer pan-genotypic SVR rates exceeding 90% in chronic HCV infection and superior tolerability compared with interferon-based regimens. Although their use is currently restricted in many countries due to high cost [13], these agents are now approved for use in acute HCV infection with treatment regimens of only eight weeks [14].

This case adds to the ongoing debate about whether screening criteria for HCV should be widened. It also highlights the importance of early recognition of HCV as a rare cause of acute hepatitis. Prompt treatment of these individuals is highly effective and has potential public health benefits in reducing the spread of the infection.

## Figures and Tables

**Table 1:** Liver function test results showing a rapid improvement in biochemical markers over a four-month period. Results displayed from day 0 (admission) to 4, 13, 30, and 120 days after admission.

Biochemical marker	Day(s)				
	0	4	13	30	120
Bilirubin (*μ*mol/L)	476	248	92	34	12
ALT (unit/L)	1,577	857	276	61	121
AST (unit/L)	847	Unavailable	80	34	51
ALP (unit/L)	177	165	130	113	101
*γ*-GT (unit/L)	Unavailable	Unavailable	47	24	24

ALT = alanine aminotransferase serum (normal 10–40); AST = aspartate aminotransferase serum (normal 15–40 unit/L); ALP = alkaline phosphatase serum (normal 30–130 unit/L); *γ*-GT = gamma-glutamyl transpeptidase serum (normal 10–66 unit/L).

**Table 2:** Viral screen, positive for active hepatitis C infection.

Test	Result
HAV total Ab	Not detected
HAV IgM	Not detected
HBsAb	>1,000 IU/L
HBsAg	Not detected
HBcAb	Not detected
HCV IgG	Detected
HCV RNA (log)	5.79 IU/mL
HCV RNA	623,706 IU/mL
HIV 1 & 2 Ab	Not detected
CMV IgG	Detected
CMV IgM	Detected
CMV DNA	<500 copies
EBV IgM	Equivocal
EBV IgG	Detected
HSV 1 & 2 DNA	Not detected
VZV DNA	Not detected

HAV = hepatitis A virus; HB = hepatitis B virus; HCV = hepatitis C virus; HIV = human immunodeficiency virus; CMV = cytomegalovirus; EBV = Epstein-Barr virus; HSV = herpes simplex virus; VZV = varicella zoster virus; Ab = antibody; Ag = antigen; “s” = surface; “c” = core; Ig = immunoglobulin.

## Additional Points

- Acute hepatitis C infection is commonly asymptomatic but should be considered in those with an acute hepatitis, particularly in the presence of risk factors.
- Novel direct-acting antiviral medications have transformed the treatment of HCV in recent years, but SVR rates for acute HCV with conventional interferon-based therapy are high (>90%).
- The advent of PrEP for HIV may change sexual practices and place patients at risk of other blood-borne viruses, which may necessitate changes in the screening strategies for HCV in the MSM population.
